# Incompatibles

**Published:** 1890-01-18

**Authors:** 


### THERAPEUTICS.

### INCOMPATIBLES.

We observe a statement in "Le Progrès Medical," to the effect that antipyrin and chloral hydrate are incompatible. Four grammes of antipyrin and five of chloral in fifteen grammes of water forms an oily precipitate, recalling somewhat the taste of coriander seed, and resembling neither chloral nor antipyrin. In the "Extra Pharmacopœia" of Martindale and Westcott, it is merely mentioned that antipyrin is incompatible with spirit of nitrous ether. Ringer, in his "Handbook of Therapeutics," does not mention incompatibility. And the "British Pharmacopœia," being, of course, behind the times, does not mention antipyrin at all. A mixture of antipyrin and quinine is also incompatible, both substances being at once precipitated from the solution.

